# Risk Assessment of Lead and Cadmium in Some Food Supplements Available on the Romanian Market

**DOI:** 10.3390/foods10030581

**Published:** 2021-03-10

**Authors:** Gabriel Mustatea, Elena L. Ungureanu, Sorin C. Iorga, Denisa Ciotea, Mona E. Popa

**Affiliations:** 1 National Research & Development Institute for Food Bioresources, 020323 Bucharest, Romania; gabi.mustatea@bioresurse.ro (G.M.); corneliu.iorga@bioresurse.ro (S.C.I.); 2 Faculty of Biotechnology, University of Agronomic Science and Veterinary Medicine, 011464 Bucharest, Romania; denisa.ciotea@gmail.com (D.C.); monapopa@agral.usamv.ro (M.E.P.)

**Keywords:** lead, cadmium, risk assessment

## Abstract

Across the world, there has been an increase in the consumption of food supplements. This may be due to the perception that these dietary supplements cannot negatively affect the health of consumers in any way. However, dietary supplements may not have the expected effect. In addition, it has been shown that supplements can sometimes be chemically, physically or microbiologically contaminated, if the hygiene conditions and Hazard Analysis Critical Control Points (HACCP) plan are not fully complied with. The aim of this study was to determine the lead and cadmium content of 41 food supplements available on the Romanian market and to assess the risk to consumer health. The graphite furnace atomic absorption spectrometry (GFAAS) technique was used for sample analysis after wet mineralization of the samples. The risk assessment was determined based on Estimated Daily Intake (EDI), Target Hazard Quotient (THQ), and Carcinogenic Risk (CR) calculations. Values obtained for both lead and cadmium fall within the limits imposed by the legislation in force. Small risks associated with the consumption of these food supplements was shown, with a main recommendation being to decrease the recommended daily dose in the case of food supplements.

## 1. Introduction

Over the years, the pharmaceutical industry has been among the most stable industries in the world [1], with a global market estimated at over 123 billion USD in 2019 [2]. This is due to the growing trend among the population of consuming various dietary supplements [3], because many consumers consider them safer and more effective compared with conventional medicines [4], being without adverse effects, even if expected effects are not achieved [3].

Food supplements are available in pharmacies and drugstores without any prescription as in the case of medicines [4]. These supplements may be named differently depending on the region and regulated by different authorities. In the US, they are called Dietary Supplements, being monitored by the Food and Drug Administration (FDA); in Canada, they are called Natural Health Products, monitored by Health Canada (HC); in Europe, they are called Food Supplements, monitored by the European Food Safety Authority (EFSA); in Australia, they are controlled by the Therapeutic Goods Administration (TGA); and in China, by the China Food and Drug Administration (CFDA) [4,5,6].

In a study conducted in the US, it was found that 70% of the elderly use dietary supplements, especially mineral-based ones [7]. Among them, women have a higher tendency to use them compared to men [7,8], but there are also studies where no differences between the sexes were observed [9]. Regarding the reason why this group of people use these supplements, the respondents ranked first the improvement of health, the health of the bones, the maintenance of the general health, and in the last place, the supplementation of the diet [9]. Among this age group, there was an increased trend for older people with higher incomes and high educational level to use dietary supplements.

According to a previous study [10], regarding the intake of dietary supplements among consumers, the consumption trend of different age groups was shown; consumers aged 51–70 years had higher intakes of vitamins A, C, and E, while consumers aged over 71 years had a higher intake of copper, magnesium, and vitamins B6 and K. 

Regarding students’ behavior, according to another study [9], 1.7% of students consumed food supplements twice a day, 41.8% consumed them daily, and the rest had a consumption frequency of 1–6 times a week. Also, among this group of consumers, multivitamins were the most frequently consumed supplements, with weight loss supplements ranking last, and more than 68% of consumers used more than one type of supplement. Regarding the source of influence, half of the respondents claimed that they bought the supplements on a doctor’s recommendation, about 15% on the recommendation of a sports coach, and lastly on the recommendation of a friend/coworker, from the internet, parents, or pharmacists [9].

However, these supplements may not always be safe, as many cases of supplements with a certain degree of contamination, especially heavy metals, have been presented in the literature [11,12]. 

Medicinal plants used as raw material in obtaining food supplements can be frequently contaminated with heavy metals due to the environment in which they are grown, being contaminated in turn with heavy metals from industry, traffic emissions, or agriculture [3]. In addition to the contamination of the raw material, the contamination of the supplements can also be caused accidentally by cross-contamination on the technological flow or by transfer from the packaging used in the product [13]. 

Excessive intake of heavy metals is dangerous for humans and animals, having carcinogenic, teratogenic, and mutagenic effects. Studies have shown that the bioaccumulation of these metals can affect various systems or organs, such as metabolic problems, diseases of the circulatory system, kidney dysfunction, diseases of the circulatory system, and increased risk of abortion. It has been shown that more than 95% of the heavy metals ingested daily comes from contaminated food [14,15]. 

The hype over dietary supplements continues to drive their consumption upwards. Thus, special attention must be accorded to the abuse and misuse of food supplements. Despite the fact of having acceptable safety profiles, many food supplements are associated with adverse effects, such as abdominal pain, agitation/irritability, tachycardia, vomiting, headache, etc. [16,17,18]. One of the most common categories of people consuming and abusing supplements is athletes, who are looking to improve their performance, to manage pain, or to reduce inflammation. When talking about improving performance, we generally talk about the doping (intentional use by the athletes of drugs or methods aimed at obtaining an improved sports performance beyond the limits possible only with training). There are many studies related to this subject, highlighting the potential health hazards caused by doping [19,20,21,22,23,24]. The use of dietary supplements among adolescents raises concerns because of differences between children and adults and the potential for the presence of additives or adulterants in dietary supplement products [16,25,26]. Regarding adolescents, differences between sexes were evident, with males being more likely to abuse dietary supplements [16]. 

The aim of the work was to evaluate the heavy metal content (lead and cadmium) in different food supplements or plant materials available on the Romanian market and to establish if these supplements comply with the law in force. Also, based on the results obtained, a risk analysis was performed on the heavy metals that can be ingested through these dietary supplements.

## 2. Materials and Methods

The analyzed samples were purchased from various drugstores or health food stores in Bucharest, Romania, in 2018 and 2019, totaling 41 food supplements of vegetal (SV1–SV30), mineral (SM1–SM9), and animal (SA1–SA2) origin, presented in Table 1. All reagent used were of analytical grade. Ultrapure nitric acid (HNO_3_ 65%) and hydrogen peroxide (H_2_O_2_) were purchased from Merck (Merck Co., Darmstadt, Germany). Calibration curves were obtained using a lead standard solution of 1000 mg/L Pb for atomic absorption (AA) (Pb(NO_3_)_2_ in 2% HNO_3_) and a cadmium standard solution of 1000 mg/L Cd for AA (Cd(NO_3_)_2_ in 2% HNO_3_) purchased from Scharlau (Scharlab, Barcelona, Spain). All dilutions were performed using ultrapure water (resistivity of 18.2 MΩ·cm), and all glassware used was cleaned and decontaminated with 10% HNO_3_.

### 2.1. Sample Preparation

To determine the content of heavy metals, 0.5 g of homogenized and crushed sample was used, which was transferred to Polytetrafluoroethylene (PTFE) dishes with 5 mL HNO_3_ and 1 mL H_2_O_2_ and subjected to mineralization according to the program presented in Table 2. The solution obtained after mineralization was quantitatively transferred to a 50 mL volumetric flask and made up to the mark, and then the samples were analyzed by GFAAS. All samples were analyzed in triplicate. 

### 2.2. Equipment

Lead and cadmium content were analyzed using an AAnalyst 600 Graphite Furnace Atomic Absorption Spectrometer system (Perkin Elmer Inc., Waltham, USA), provided with a Transversely Heated Graphite Atomizer (THGA) furnace assembly, longitudinal Zeeman-effect background correction, enhanced STPF technology, and True Temperature Control (TTC) to provide an unmatched graphite furnace AA performance. For the wet digestion of the samples, an MWS-2 Berghof (Berghof Products + Instruments GmbH, Eningen, Germany) type microwave mineralizer was used, provided with 5 mineralization vessels.

A calibration curve for each element was established using the reagents described above, consisting of 5 points: 10 µg·L^−1^, 20 µg·L^−1^, 30 µg·L^−1^, 40 µg·L^−1^, 50 µg·L^−1^. The calibration curve for both the elements revealed a good linearity over the whole range of concentrations. The accuracy of the method was assessed by testing the SRM 3280 reference material in triplicate. Recovery for lead and cadmium in SRM 3280 was higher than 95.0%. For both elements, the detection limit was calculated by analyzing the first standard in 10 replicates, obtaining a detection and quantification limit, for Pb, of 1.2 µg·L^−1^ and 3.6 µg·L^−1^, respectively, and for Cd, of 0.2 µg·L^−1^ and 0.6 µg·L^−1^, respectively. The regression coefficient for both metals was higher than 0.995.

### 2.3. Human Health Risk Assessment

The model used by Romero-Estevez et al. [14] was used to assess the risk of contamination with heavy metals from food supplements on human health. For this, Estimated Daily Intake (EDI), Target Hazard Quotients (THQ), and Carcinogenic Risk (CR) were calculated. These parameters were established for both metals (lead and cadmium) for a person of 70 kg and age of 70 years, considering that these dietary supplements are not recommended for children. The daily supplement intake was calculated based on the recommended dose mentioned on the food supplement label.

The Estimated Daily Intake, expressed in µg·kg^−1^·day^−1^, was calculated for each metal, using the following Equation (1), recommended by the US EPA [27]:

EDI = C × IR × EF × ED/BW × AT(1)

where C is the metal concentration in the sample in µg·kg^−1^ for solid samples and µg·L^−1^ for liquid samples (tinctures), IR is the ingestion rate (calculated based on the recommended dose) in mg/day or mL/day (for tinctures), EF is the exposure frequency (365 days per year), ED is the exposure duration (70 years), BW is the body weight (70 kg), and AT represents the average exposure time (EF × ED).

Based on Equation (1), the Target Hazard Quotient (THQ) was calculated for non-carcinogenic risk. This parameter was established for each metal according to the following Equation (2), according to the US EPA [27]:

THQ = EDI/RfD(2)

where EDI is the Estimated Daily Intake, in µg·kg^−1^·day^−1^, and RfD is the Reference Dose, in mg·kg^−1^·day^−1^, which represents the tolerable daily intake of the metal via oral exposure. The RfDs of lead and cadmium are 3.5 · 10^−3^ mg·kg^−1^·day^−1^ and 1 · 10^−3^ mg·kg^−1^·day^−1^, respectively [28].

In this study, the Total Cumulative Health Risk (TTHQ) was calculated by adding the THQ values obtained for each metal, according to Equation (3). A higher value of TTHQ is an important cause for concern, as it can lead to adverse health effects [14].

TTHQ = THQ_(Pb)_ + THQ_(Cd)_ (3)

The Carcinogenetic Risk (CR) of carcinogenic effects is a person’s likelihood of developing cancer during his or her lifetime due to exposure to metals. To establish this parameter, a slope factor is needed, which is available only for lead. There is currently no value for this parameter for cadmium.

The calculation of this parameter was performed according to Equation (4).

CR = CSF × EDI(4)

where CSF is the Cancer Slope Factor in mg·kg^−1^·day^−1^ and EDI is Estimated Daily Intake in µg·kg^−1^·day^−1^. The Cancer Slope Factors for lead and cadmium are 0.0085 mg·kg^−1^·day^−1^ [29] and 0.38 mg·kg^−1^·day^−1^ [30], respectively.

### 2.4. Data analysis

All experiments were conducted in triplicate, and results were expressed as means. A one-way analysis of variance ANOVA was computed with the SPSS statistics program to assess if there were significant differences between the means for both heavy metals: lead and cadmium. It was also investigated if the origin of these supplements influenced the measurement error. 

## 3. Results and Discussion

Lead and cadmium contents found in different food supplements are presented in Table 3. The results found were compared with EU Regulation 1881/2006 [31], which imposes a maximum allowed limit of 3.0 mg/kg for lead and 1.0 mg/kg for cadmium.

As can be seen from Table 3, the values obtained for the two metals did not exceed the limits imposed. It can also be seen that lead and cadmium were found in 68% of the samples.

As a first conclusion, it can be seen that in the case of food supplements obtained from plants, the values obtained were higher compared to food supplements of mineral or animal origin.

This can be supported by the maximum concentration found of 1274 mg/kg in the case of lead for the sample SV26, a food supplement obtained from various plants. The highest concentration of cadmium was also found in a sample of plant origin (sample SV13), namely 0.203 mg/kg.

### Human Health Risk Assessment

For this study, the daily consumption of the tested food supplements was taken into account, at the maximum doses indicated on the label of each product.

Table 4 and Table 5 show the values obtained for EDI, THQ, and TTHQ for the two heavy metals analyzed.

As can be seen in Table 4, the EDI values varied between 0 and 5.460 µg·kg^−1·^day^−1^, and the THQ between 0 and 1820.

If the THQ value is lower than 1 (one), consumers may not experience adverse health effects, but if THQ is greater than or equal to 1, then consumers are exposed to a potential risk [14].

In the case of lead, the highest values were found in the food supplements of plant origin, where the highest values of this metal were also found.

Also, the highest values of EDI (5.460 µg·kg^−1^·day^−1^) and THQ (1.820) were obtained in the case of sample SV26, where the highest amount of lead was found. Because the THQ > 1, a decrease in the recommended daily intake stated on the product label may be recommended. THQ values higher than 1 can be observed in the case of SV29 and SV21 samples, namely 1.38 and 1.128, respectively.

Important values of EDI were also found in the case of food supplements of mineral origin; although without the THQ being higher than 1, for these samples, the maximum recommended doses were in the order of grams of product per day.

In the case of cadmium, the EDI and THQ values varied between 0 and 1.143 µg·kg^−1^·day^−1^. The highest value found was for the SA1 sample, most likely due to the recommended daily dose that is quite high, because the amount of cadmium did not exceed the required value. It may be recommended to decrease the recommended daily dose for this dietary supplement.

As in the case of lead, important values of EDI and THQ were obtained for cadmium for food supplements of mineral origin, due to a high recommended dose. With the exception of SV29, this time, very low values of EDI and THQ were obtained in the samples of plant origin, because the cadmium concentrations found were quite low.

Table 4 shows the TTHQ values for the two metals analyzed for all 41 samples. The values obtained varied between 0 and 1854. This parameter was obtained by summing the THQ values for each metal, which are calculated for an average person of 70 kg and 70 years of age. TTHQ values less than or equal to 1 indicate that exposure does not cause side effects, and values greater than 1 do not necessarily suggest a high probability of producing some side effects on consumer health [32].

The Carcinogenic Risk (CR) was calculated for all samples for both lead and cadmium. Values of this parameter less than 1E-6 are considered tolerable, those between 1E-6 and 1E-4 are considered in the acceptable range, and those greater than 1E-4 are considered intolerable [14].

According to the results presented in Table 5, it can be seen that the Carcinogenic Risk due to the ingestion rate varied between 0 and 4.60E-2 for lead and between 0 and 4.34E-1 for cadmium.

In the case of Pb, 31.7% of tested samples were considered tolerable, 19.5% within the acceptable range, and 48.8% intolerable. Regarding Cd, 56% of the samples were considered intolerable, 44% tolerable, and no values were obtained that were within the tolerable range.

The results for the CR for lead and cadmium showed that 24% of samples were tolerable, 19.5% were in the tolerable range, and 56.1% were intolerable. As can be seen, more than half of the samples analyzed had a carcinogenic risk in terms of lead and cadmium content, which may be considered a matter of concern.

Among the studied heavy metals, cadmium had the highest chance of posing a cancer risk (mean CR 3.77E-3), while lead had the lowest chance of posing a cancer risk (mean CR 2.37E-5).

Referring to the total CR, consisting of CR_Pb_ and CR_Cd_ (Table 5), the acceptable level is 10^−5^ [33]. In this case, it can be seen that only samples SV3 and SV10 were considered tolerable, and the rest were in the tolerability range or intolerable.

It can be seen that most of the samples considered intolerable in terms of CR belonged to food supplements of plant origin, which emphasizes once again the degree of contamination with heavy metals that can have certain carcinogenic effects on consumers. A measure to protect consumers of these supplements may be the reduction of the recommended daily dose. 

**Figure 1:**
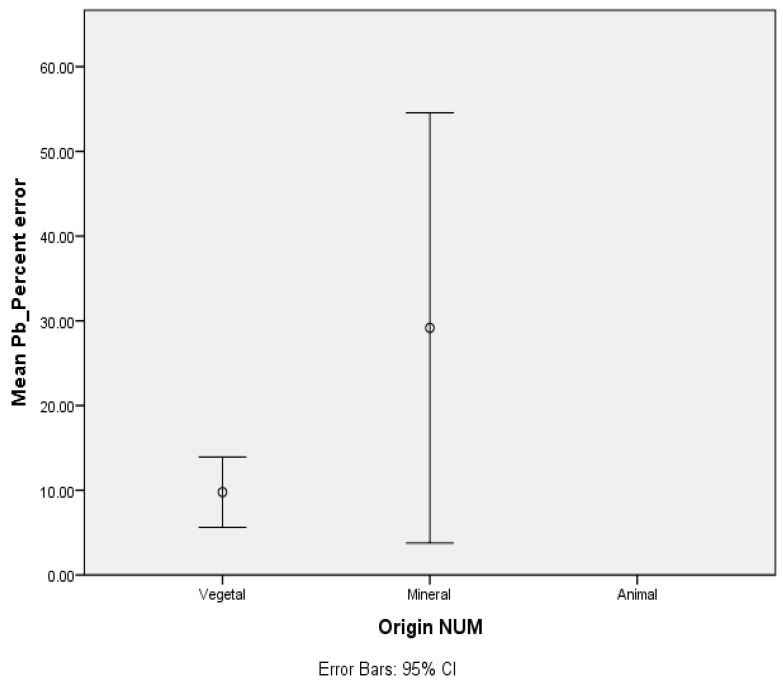
Mean error for Pb analysis.

The one-way ANOVA results presented in Figure 1 indicate that the level of measurement of mean error for Pb determinations were influenced by the origin of the product. Apparently, the mean error margin was higher for mineral products.

In Table 6, it is also shown that the means for EDI_Cd_, THQ_Cd_, CR_Cd_, and CR_Pb+Cd_ were influenced by the origin of the product. 

As presented in Table 7, a comparison was made with data from the literature, in terms of lead and cadmium contents in various food supplements of vegetal origin. 

According to the results in Table 7, it can be seen that the differences that occur between different plant raw materials from which food supplements are derived may be due to several factors, such as the area in which they are grown [37] and the part of the plant which is used to obtain various supplements [34], but also others, such as the degree of maturation of the plant, the harvest season, and the method of analysis. 

Regarding the consumption of food supplements, certain recommendations can be mentioned, both for the consumer and for the producer, such as informing the consumer before buying a food supplement; avoiding products presented as “miraculous”, which have only benefits without side effects; avoiding taking more than two or three food supplements at the same time, because in the case of mixtures of ingredients, some side effects may occur; taking supplements during pregnancy only with a doctor’s recommendation; taking food supplements only in the recommended doses and not exceeding the indicated administration period on the label [38]. 

## 4. Conclusions

For both lead and cadmium, the results obtained did not exceed the limits of 3.0 mg/kg and 1.0 mg/kg, respectively, imposed by EU Regulation no. 1881/2006.

It can also be seen that in the samples of tea, the values obtained were comparatively higher. These results may be influenced by soil conditions, harvest season, the degree of maturity of the leaves at harvest, and the method of analysis.

Regarding the risk assessment of ingestion of these metals, it was observed that the highest values of EDI, THQ, TTHQ, and CR were found in the case of samples of plant origin. This aspect is also influenced by the dose recommended by the producer, which, in order to avoid ingesting a large quantity of heavy metals through dietary supplements, can be reduced so as not to endanger the health of consumers. More detailed studies are also needed to highlight this.

## Figures and Tables

**Table 1:** Description of samples analyzed for heavy metal content.

Code	Sample	Presentation and Packaging	Origin
SV1	Red cranberry tincture	Liquid in brown glass	Vegetal
SV2	Blueberry tincture	Liquid in brown glass	Vegetal
SV3	Sea buckthorn tincture	Liquid in brown glass	Vegetal
SV4	Chimney tincture	Liquid in brown glass	Vegetal
SV5	Marigold tincture	Liquid in brown glass	Vegetal
SV6	Propolis tincture	Liquid in brown glass	Vegetal
SV7	Elderflower tincture	Liquid in brown glass	Vegetal
SV8	Sea buckthorn fruits (dried)	100 g dried fruit in a paper bag with a PP window	Vegetal
SV9	Rosehip fruits (dried)	100 g dried fruit in a paper bag with a PP window	Vegetal
SV10	Blueberry fruits (dried)	100 g dried fruit in a paper bag with a PP window	Vegetal
SV11	Herbal tea 1	20 tea bags in a cardboard box	Vegetal
SV12	Herbal tea 2	20 tea bags in a cardboard box	Vegetal
SV13	Herbal tea 3	20 tea bags in a cardboard box	Vegetal
SV14	Mint tea 1	20 tea bags in a cardboard box	Vegetal
SV15	Mint tea 2	20 tea bags in a cardboard box	Vegetal
SV16	Mint tea 3	20 tea bags in a cardboard box	Vegetal
SV17	Shredded linden flowers	Liquid in brown glass	Vegetal
SV18	Marigold flowers	100 g dried fruit in a paper bag with a PP window	Vegetal
SV19	Thyme	100 g dried fruit in a paper bag with a PP window	Vegetal
SV20	Red clover	Liquid in brown glass	Vegetal
SV21	Echinacea	60 tablets in a cardboard box	Vegetal
SV22	Food supplement for digestion	60 tablets in a cardboard box	Vegetal
SV23	Food supplement for the nervous system	60 tablets in a cardboard box	Vegetal
SV24	Food supplements for immunity	60 tablets in a cardboard box	Vegetal
SV25	Food supplement for the immune system	60 tablets in a cardboard box	Vegetal
SV26	Herbal antioxidant	60 tablets in a cardboard box	Vegetal
SV27	Sea buckthorn syrup with honey	200 mL syrup in a brown bottle	Vegetal
SV28	Peeled hemp seeds	100 g dried fruit in a paper bag with a PP window	Vegetal
SV29	Cold-pressed hemp oil	200 mL oil in a brown bottle	Vegetal
SV30	L-carnitine	40 tablets in a cardboard box	Vegetal
SM1	Mineralized zeolite	60 capsules in a white vial	Mineral
SM2	Mineralized zeolite	60 capsules in a blue vial	Mineral
SM3	Activated zeolite	60 capsules in a white vial	Mineral
SM4	Active zeolite	30 capsules in a white vial	Mineral
SM5	Active zeolite	60 capsules in a blue vial	Mineral
SM6	Natural zeolite	60 capsules in a white vial	Mineral
SM7	Zeolite dry powder	350 g powder in a white vial	Mineral
SM8	Zeolite powder	125 g powder in a white vial	Mineral
SM9	Clay powder	200 clay powder in laminated paper	Mineral
SA1	Omega 3	60 capsules in a green vial	Animal
SA2	Protein concentrate powder	500 g casein protein powder in a laminated bag	Animal

PP—polypropylene

**Table 2:** Microwave heating program.

Step	Temperature (°C)	Duration (min)	Power (%)
1	160	5	80
2	220	40	90
3	cooling	20	0

**Table 3:** Heavy metal content in analyzed samples.

Sample	Pb (mg/kg)	Cd (mg/kg)
SV1	0.010 ± 0.001	0.0006 ± 0.0001
SV2	0.341 ± 0.015	<0.0002
SV3	0.004 ± 0.001	0.0005 ± 0.0002
SV4	0.323 ± 0.013	<0.0002
SV5	0.003 ± 0.001	0.0006 ± 0.0001
SV6	0.026 ± 0.009	0.0007 ± 0.0001
SV7	<0.002	0.0004 ± 0.0001
SV8	<0.002	<0.0002
SV9	<0.002	<0.0002
SV10	0.009 ± 0.002	<0.0002
SV11	0.074 ± 0.004	0.1930 ± 0.0010
SV12	0.080 ± 0.002	0.1740 ± 0.0012
SV13	0.151 ± 0.003	0.2030 ± 0.0009
SV14	0.643 ± 0.011	0.0350 ± 0.0009
SV15	0.146 ± 0.008	0.0070 ± 0.0003
SV16	0.135 ± 0.007	0.0220 ± 0.0010
SV17	0.253 ± 0.011	0.1030 ± 0.0015
SV18	0.530 ± 0.014	0.0040 ± 0.0001
SV19	0.174 ± 0.008	0.0540 ± 0.0006
SV20	<0.002	<0.0002
SV21	0.191 ± 0.006	0.0020 ± 0.0002
SV22	0.042 ± 0.003	0.0005 ± 0.0001
SV23	0.012 ± 0.002	0.0021 ± 0.0003
SV24	0.012 ± 0.001	<0.0002
SV25	0.013 ± 0.002	<0.0002
SV26	1.274 ± 0.013	0.0040 ± 0.0002
SV27	<0.002	0.0009 ± 0.0001
SV28	<0.002	0.0360 ± 0.0009
SV29	0.323 ± 0.010	0.0069 ± 0.0004
SV30	0.032 ± 0.004	0.0005 ± 0.0001
SM1	0.002 ± 0.001	<0.0002
SM2	< 0.002	0.0480 ± 0.0008
SM3	<0.002	<0.0002
SM4	0.003 ± 0.001	0.0200 ± 0.0005
SM5	0.006 ± 0.001	<0.0002
SM6	<0.002	<0.0002
SM7	<0.002	0.0008 ± 0.0001
SM8	0.012 ± 0.002	0.0110 ± 0.0003
SM9	<0.002	0.0480 ± 0.0006
SA1	<0.002	0.0400 ± 0.0009
SA2	<0.002	<0.0002

**Table 4:** Estimated Daily Intake (EDI), Target Hazard Quotients (THQ), and Total Cumulative Health Risk (TTHQ) for lead and cadmium from food supplements consumed by adults.

Sample	EDI_Pb_ µg·kg^−1^·day^−1^	THQ_Pb_	EDI_Cd_	THQ_Cd_	TTHQ
SV1	0.020	0.007	0.001	0.001	0.008
SV2	0.317	0.106	NA	NA	0.106
SV3	0.001	NA	NA	NA	NA
SV4	0.485	0.162	NA	NA	0.162
SV5	0.003	0.001	0.001	0.001	0.002
SV6	0.024	0.008	0.001	0.001	0.009
SV7	NA	NA	NA	NA	NA
SV8	NA	NA	NA	NA	NA
SV9	NA	NA	NA	NA	NA
SV10	0.001	NA	NA	NA	NA
SV11	0.005	0.002	0.014	0.014	0.016
SV12	0.006	0.002	0.012	0.012	0.014
SV13	0.011	0.004	0.015	0.015	0.019
SV14	0.046	0.015	0.003	0.003	0.018
SV15	0.010	0.003	0.001	0.001	0.004
SV16	0.010	0.003	0.002	0.002	0.005
SV17	0.018	0.006	0.007	0.007	0.013
SV18	0.038	0.013	NA	NA	0.013
SV19	0.012	0.004	0.004	0.004	0.008
SV20	NA	NA	NA	NA	NA
SV21	3.383	1.128	0.053	0.053	1.181
SV22	0.648	0.216	0.008	0.008	0.224
SV23	0.086	0.029	0.015	0.015	0.044
SV24	0.720	0.240	NA	NA	0.240
SV25	0.065	0.217	NA	NA	0.217
SV26	5.460	1.820	0.034	0.034	1.854
SV27	NA	NA	NA	NA	NA
SV28	NA	NA	0.015	0.015	0.015
SV29	4.614	1.538	0.296	0.296	1.834
SV30	0.206	0.069	0.003	0.003	0.072
SM1	0.103	0.034	NA	NA	0.034
SM2	NA	NA	0.314	0.314	0.314
SM3	NA	NA	NA	NA	NA
SM4	0.206	0.069	NA	NA	0.069
SM5	0.309	0.103	NA	NA	0.103
SM6	NA	NA	NA	NA	NA
SM7	NA	NA	0.114	0.114	0.114
SM8	1.371	0.457	0.157	0.157	0.614
SM9	NA	NA	0.343	0.343	0.343
SA1	NA	NA	1.143	1.143	1.143
SA2	NA	NA	NA	NA	NA

NA—not applicable.

**Table 5:** Carcinogenetic Risk (CR) values for lead and cadmium.

Sample	CR_Pb_	CR_Cd_	CR_Pb+Cd_
SV1	1.70E-4	3.80E-4	5.50E-4
SV2	2.69E-3	NA	2.69E-3
SV3	8.50E-6	NA	8.50E-6
SV4	4.12E-3	NA	4.12E-3
SV5	2.60E-5	3.80E-4	4.06E-4
SV6	2.00E-4	3.80E-4	5.84E-4
SV7	NA	NA	NA
SV8	NA	NA	NA
SV9	NA	NA	NA
SV10	8.50E-6	NA	8.50E-6
SV11	4.30E-5	5.32E-3	5.36E-3
SV12	5.10E-5	4.56E-3	4.61E-3
SV13	9.40E-5	5.70E-3	5.79E-3
SV14	3.90E-4	1.14E-3	1.53E-3
SV15	8.50E-5	3.80E-4	4.65E-4
SV16	8.50E-5	7.60E-4	8.45E-4
SV17	1.50E-4	2.66E-3	2.81E-3
SV18	3.20E-4	NA	3.23E-4
SV19	1.00E-4	1.52E-3	1.62E-3
SV20	NA	NA	NA
SV21	2.88E-2	2.01E-2	4.89E-2
SV22	5.51E-3	3.04E-3	8.55E-3
SV23	7.30E-4	5.70E-3	6.43E-3
SV24	6.12E-3	NA	6.12E-3
SV25	5.50E-4	NA	5.53E-4
SV26	4.65E-2	1.29E-2	5.93E-2
SV27	NA	NA	NA
SV28	NA	5.70E-3	5.70E-3
SV29	3.92E-2	1.12E-1	1.52E-1
SV30	1.75E-3	1.14E-3	2.89E-3
SM1	8.80E-4	NA	8.76E-4
SM2	NA	1.19E-1	1.19E-1
SM3	NA	NA	NA
SM4	1.75E-3	NA	1.75E-3
SM5	2.63E-3	NA	2.63E-3
SM6	NA	NA	NA
SM7	NA	4.33E-2	4.33E-2
SM8	1.17E-2	5.97E-2	7.13E-2
SM9	NA	1.30E-1	1.30E-1
SA1	NA	4.34E-1	4.34E-1
SA2	NA	NA	NA

NA—not applicable.

**Table 6:** One-way ANOVA results (test between means).

ANOVA						
		Sum of Squares	df	Mean Square	F	Sig.
EDI_Pb_	Between groups	0.108	1	0.108	0.052	0.822
	Within groups	53.998	26	2.077		
	Total	54.106	27			
THQ_Pb_	Between groups	0.026	1	0.026	0.109	0.744
	Within groups	5.839	24	0.243		
	Total	5.865	25			
EDI_Cd_	Between groups	1.251	2	0.625	105.749	0.000
	Within groups	0.118	20	0.006		
	Total	1.369	22			
THQ_Cd_	Between groups	1.251	2	0.625	105.749	0.000
	Within groups	0.118	20	0.006		
	Total	1.369	22			
TTHQ	Between groups	0.769	2	0.385	1.527	.235
	Within groups	7.052	28	0.252		
	Total	7.821	30			
CR_Pb_	Between groups	0.000	1	0.000	0.051	0.823
	Within groups	0.004	26	0.000		
	Total	0.004	27			
CR_Cd_	Between groups	0.180	2	0.090	106.500	0.000
	Within groups	0.017	20	0.001		
	Total	0.197	22			
CR_Pb+Cd_	Between groups	0.174	2	0.087	59.490	0.000
	Within groups	0.044	30	0.001		
	Total	0.217	32			
Pb_Percent error	Between groups	1288.604	1	1288.604	11.207	0.002
	Within groups	2989.406	26	114.977		
	Total	4278.010	27			
Cd_Percent error	Between groups	211.575	2	105.788	1.184	0.323
	Within groups	2233.580	25	89.343		
	Total	2445.155	27			

**Table 7:** Heavy metals concentrations (mg/kg) in different food supplements.

Reference	Pb	Cd	Method Used
[4]	<0.005	<0.010	ICP-OES
[34]	0.00–0.015	0.00–0.099	ICP-AES
[35]	0.220–1.632	0.014–0.163	ICP-MS
[36]	<1.000–23.520	0.100–1.110	GFAAS
[37]	1.100–195.900	0.200–0.016	GFAAS
This study	<0.002–1.274	< 0.0002–0.2030	GFAAS

ICP-OES, Inductively Coupled Plasma Optical Emission Spectrometry; GFAAS, Graphite Furnace Atomic Absorption Spectrometry; ICP-AES, Inductively Coupled Plasma Atomic Emission Spectrometry; ICP-MS, Inductively Coupled Plasma Mass Spectrometry.
